# Supplementary material for: Hepatitis C Virus Treatment Status and Barriers among Patients in Methadone Maintenance Treatment Clinics in Guangdong Province, China: A Cross-Sectional, Observational Study
Source: Int J Environ Res Public Health. 2019 Nov 12;16(22):4436. doi: 10.3390/ijerph16224436 (PMC6888391; doi:10.3390/ijerph16224436)
Supplement: Supplementary file 1 [file ijerph-16-04436-s001.pdf]

Table S1. Patient characteristics by reported HCV status in MMT clinics, Guangdong, China.

Characteristics	Self-reported HCV status (n, (%))			*Total*
	Reporting infection (n=366)	*Reporting no infection (n=259)*	*Unknown (n=53)*	*(n=678)*
Predisposing factors				
**Gender**				
Male	322 (88.0)	230 (88.8)	47 (88.7)	599 (88.3)
Female	44 (12.0)	29 (11.2)	6 (11.3)	79 (11.7)
**Age (years)**				
18-34	32 (8.7)	24 (9.3)	3 (5.7)	59 (8.7)
34-44	202 (55.2)	113 (43.6)	23 (43.4)	338 (49.9)
≥45	132 (36.1)	122 (47.1)	27 (50.9)	281 (41.4)
$\bar{x} \pm s$	42.7±6.17	43.9±7.20	44.9±6.7	43.3±6.65
**Marital status**				
Married	219 (59.8)	166 (64.1)	31 (58.5)	416 (61.4)
Single	96 (26.2)	55 (21.2)	16 (30.2)	167 (24.6)
Divorced or windowed	51 (13.9)	38 (14.7)	6 (11.3)	95 (14.0)
**Duration in MMT (years)**				
<5	153 (41.8)	106 (40.9)	22 (41.5)	281 (41.4)
≥5	213 (58.2)	153 (59.1)	31 (58.5)	397 (58.6)
**Region of residence**				
urban	249 (68.0)	169 (65.3)	34 (64.2)	452 (66.7)
rural	117 (32.0)	90 (34.7)	19 (35.8)	226 (33.3)
**Duration of abusing drugs (years)**[#]				
<10	75 (20.5)	56 (21.6)	6 (11.3)	137 (20.2)
10~19	230 (62.8)	145 (56.0)	34 (64.2)	409 (60.3)
≥20	57 (15.6)	55 (21.2)	13 (24.5)	125 (18.4)
$\bar{x} \pm s$	14.1±5.71	14.3±6.25	15.6±5.98	14.3±5.95
missing	4 (1.1)	3 (1.2)	0 (0.0)	7 (1.0)
**Ever injected drugs**				
No	59 (16.1)	57 (22.0)	9 (17.0)	125 (18.4)
Yes	307 (83.9)	199 (76.8)	44 (83.0)	550 (81.1)
missing	0 (0.0)	3 (1.2)	0 (0.0)	3 (0.4)
**Ever shared needles**				
No	257 (70.2)	220 (84.9)	44 (83.0)	521 (76.8)
Yes	109 (29.8)	39 (15.1)	9 (17.0)	157 (23.2)
**Abusing drugs in the past month**				
No	324 (88.5)	232 (89.6)	47 (88.7)	603 (89.3)
Yes	42 (11.5)	27 (10.4)	6 (11.3)	75 (10.7)
**More than one sexual partner in the past month**				
No	348 (95.1)	240 (92.7)	48 (90.6)	636 (93.8)

Yes	18 (4.9)	19 (7.3)	5 (9.4)	42 (6.2)
Enabling factors				
**Education level**				
≤Primary school	85 (23.2)	52 (20.1)	16 (30.2)	153 (22.6)
Junior high school	205 (56.0)	152 (58.7)	30 (56.6)	387 (57.1)
≥ Senior high school	76 (20.8)	55 (21.2)	7 (13.2)	138 (20.4)
**Employment**				
Unemployed	194 (53.0)	73 (28.2)	24 (45.3)	291 (42.9)
Employed	172 (47.0)	186 (71.8)	29 (54.7)	387 (57.1)
**Monthly income**				
<3000	217 (59.3)	150 (57.9)	34 (64.2)	401 (59.1)
3000-4999	111 (30.3)	84 (32.4)	17 (32.1)	212 (31.3)
≥5000	33 (9.0)	24 (9.3)	2 (3.8)	59 (8.7)
missing	5 (1.4)	1 (0.4)	0 (0.0)	6 (0.9)
**Have health insurance**				
Yes	272 (74.3)	184 (71.0)	39 (73.6)	495 (73.0)
No	89 (24.3)	75 (29.0)	14 (26.4)	178 (26.3)
missing	5 (1.4)	0 (0.0)	0 (0.0)	5 (0.7)
**Have stable residence**				
Yes	309 (84.4)	233 (90.0)	45 (84.9)	587 (86.6)
No	57 (15.6)	26 (10.0)	8 (15.1)	91 (13.4)
Need factors				
**Drinking times in the past month**				
Never	258 (70.5)	177 (68.3)	34 (64.2)	469 (69.2)
1-3 times per month	57 (15.6)	45 (17.4)	9 (17.0)	111 (16.4)
≥1 times per week	25 (6.8)	15 (5.8)	4 (7.5)	44 (6.5)
≥1 times per day	25 (6.8)	21 (8.1)	6 (11.3)	52 (7.7)
missing	1 (0.3)	0 (0.0)	0 (0.0)	1 (0.1)
**HIV infection**				
No	313 (85.5)	241 (93.0)	49 (92.5)	603 (88.9)
Yes	53 (14.5)	15 (5.8)	4 (7.5)	72 (10.6)
missing	0 (0.0)	3 (1.2)	0 (0.0)	3 (0.4)
**HBV infection**				
No	334 (91.3)	252 (97.3)	48 (90.6)	634 (93.5)
Yes	32 (8.7)	4 (1.5)	5 (9.4)	41 (6.1)
missing	0 (0.0)	3 (1.2)	0 (0.0)	3 (0.4)

before entering in MMT. Sample size may vary due to missing data.
